# Teaching Communication as a Procedure by Utilizing a Mixed-Methods Curriculum: A Pilot Study

**DOI:** 10.7759/cureus.25597

**Published:** 2022-06-02

**Authors:** Carly Levy, Maria Carmen G Diaz, Mindy Dickerman

**Affiliations:** 1 General Pediatrics/Palliative Medicine, Nemours Children's Health/Sidney Kimmel Medical College at Thomas Jefferson University, Wilmington, USA; 2 General Pediatrics/Emergency Medicine, Nemours Children's Health/Sidney Kimmel Medical College at Thomas Jefferson University, Wilmington, USA; 3 General Pediatrics/Critical Care and Palliative Medicine, Nemours Children's Health/Sidney Kimmel Medical College at Thomas Jefferson University, Wilmington, USA

**Keywords:** medical education, fellows, procedural training, serious news, communication training

## Abstract

Objective

In this study, we aimed to develop and pilot a mixed-methods curriculum among pediatric subspecialty fellows that combined didactics, role-play, and bedside coaching with a procedure card. We hypothesized that this curriculum would improve fellows’ ability to navigate difficult conversations and would be feasible to implement across training programs.

Methods

This study was conducted from 2019 to 2020. Phase 1 focused on establishing baseline performance. Phase 2 involved the education of participants and faculty. During phase 3, participants communicated difficult news to patients and families using the procedure card as a prompt with the aid of faculty coaching. Six months later, participants' performance was re-evaluated and compared with baseline performance.

Results

A total of 10 out of 17 (60%) participants completed the pilot study. Likert self-efficacy results revealed an improvement in the skill of delivering difficult news (3.0 pre-intervention, 4.1 post-intervention, p=0.0001), conducting a family conference (2.5 pre-intervention, 3.6 post-intervention, p=0.0001), and responding to emotions (3.4 pre-intervention, 4.2 post-intervention, p=0.0003). Investigator assessments showed improvement in fellows’ ability to communicate information clearly (2.5 pre-intervention, 3.9 post-intervention, p=0.0001) and demonstrate empathy (2.7 pre-intervention, 3.3 post-intervention, p=0.005).

Conclusions

In this pilot study, coaching at the bedside with a procedure-card prompt was effective at improving specific self-perceived and observed communication skills. Future research is needed to evaluate modifications to this curriculum to enhance its feasibility.

## Introduction

Clinicians are often required to navigate difficult conversations with patients and families. To effectively lead these conversations, providers need to master the procedure of clear and compassionate communication. Family members of children and adults with serious illnesses report that effective communication informs their decision-making, improves their coping skills with respect to illness, and leads to improved quality of care [1-3]. Without proper training, providers may adopt inappropriate ways of delivering serious news, leading to emotional consequences such as fatigue and burnout [4,5].

To gain proficiency in any procedure in medicine, clinicians require education, training, and practice [6]. Although difficult conversations have not been formally defined as a "procedure" in medicine, the medical community, including the Accreditation Council of Graduate Medical Education and other medical subspecialty societies, recognizes communication training as a priority [7,8]. Several communication training methods have been studied in different clinician populations: simulation, role-play, the use of cognitive maps, checklists, as well as deliberate practice and feedback have all been shown to improve performance or self-efficacy in navigating difficult conversations [9-19]. Specifically, the VitalTalk [20] curriculum enhances communication skills while increasing the frequency of empathic behaviors [21,22]. Although many studies over the last decade have looked at strategies to teach communication skills, further research is needed to define the best methods to ensure feasibility and reproducibility within the learners’ work environment, as well as skill retention over time [19,23,24].

This pilot study aimed to develop a specific intervention to improve pediatric critical care, neonatology, and hematology/oncology fellows’ ability to deliver serious news. The pilot curriculum combined didactics, role-play, and bedside coaching by local faculty with the aid of a procedure card. The procedure card provided trainees with stepwise guidance for the procedure of delivering difficult news by adapting one of VitalTalk’s [20] well-established cognitive maps (used with permission). The card also included a feedback tool for the faculty. The intention of the procedure card was to serve as a deliberate prompt for learners to be reminded of the steps and skills required to deliver difficult news as well as a prompt for the faculty to provide feedback following the encounter. We hypothesized that this curriculum would improve fellows’ ability to navigate difficult conversations and would be feasible to implement across subspecialty training programs.

This article was presented virtually as a poster at the Pediatric Academic Society in May 2021 and the Association of Pediatric Program Directors in March 2021.

## Materials and methods

This study was conducted from 2019 to 2020 at a free-standing children’s hospital and was approved by the local Institutional Review Board. All first-year and second-year pediatric critical care, neonatology, and hematology/oncology fellows were invited to participate in the study via email. Participation was voluntary. This prospective educational intervention consisted of four phases:

Phase 1: establishing a baseline performance

After enrollment, participants completed a self-efficacy survey that entailed reporting their prior training, experience, and confidence in delivering difficult news. The survey was developed iteratively through a review of previously published self-efficacy surveys [10,25]. The survey was reviewed by content experts in palliative care, communication, and medical education to ensure face validity. The survey used a 1-5 Likert scale with 1 indicating that the participant did not feel well-prepared and 5 indicating that the participant felt very well-prepared. Following the survey, each participant took part in a simulation scenario focused on communicating difficult news to a parent, who was portrayed by a trained actor. Study investigators watched a live stream of the simulation and rated each participant’s performance and communication skills. Additionally, the actor rated each participant. Study investigators followed each simulation session with a debrief, which served as an introduction to key skills taught in the curriculum.

Phase 2: directed education for participants and faculty

**Figure 1:**
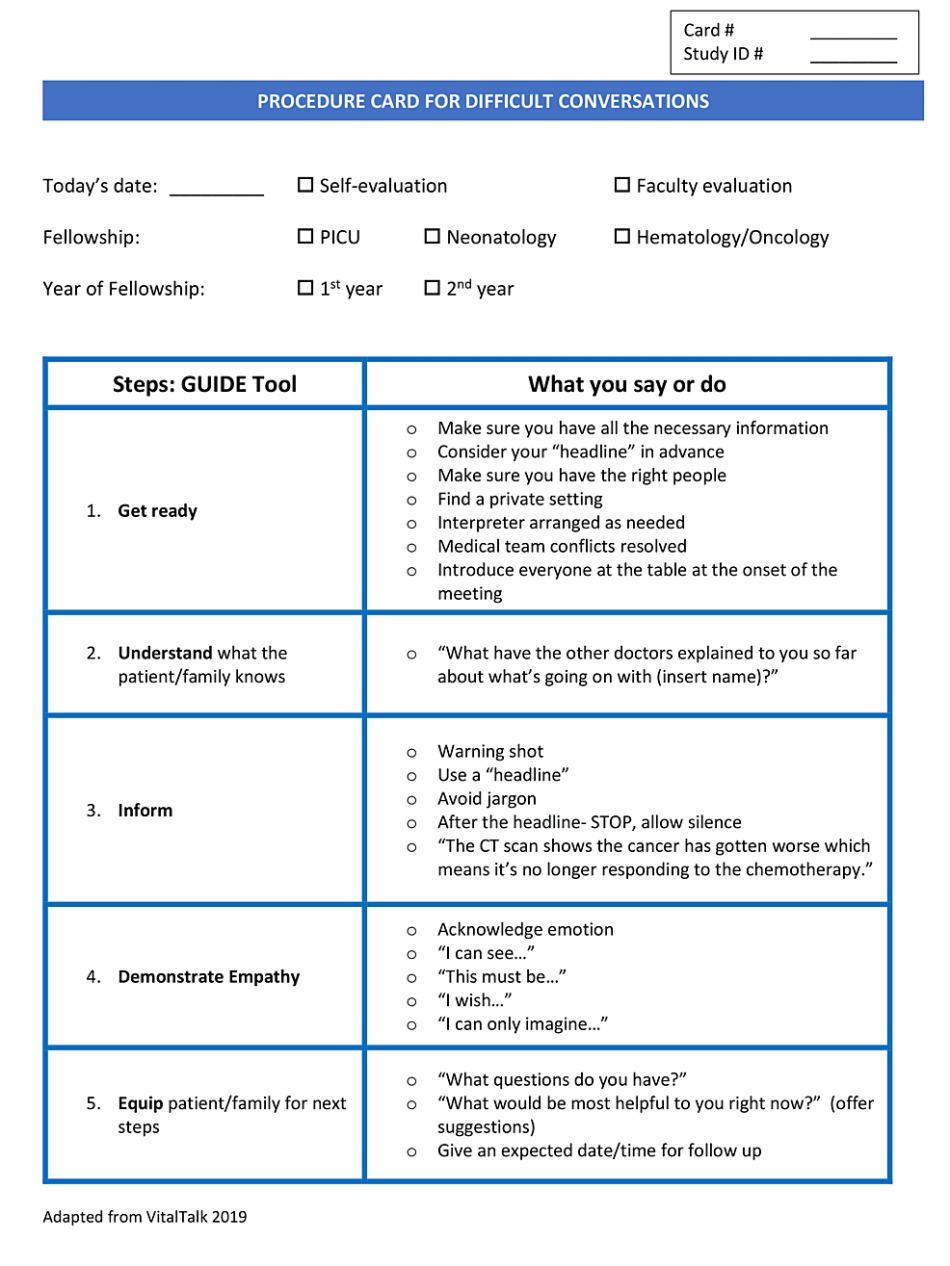
Procedure card for delivering serious news (side A) Adapted from VitalTalk [20]

**Figure 2:**
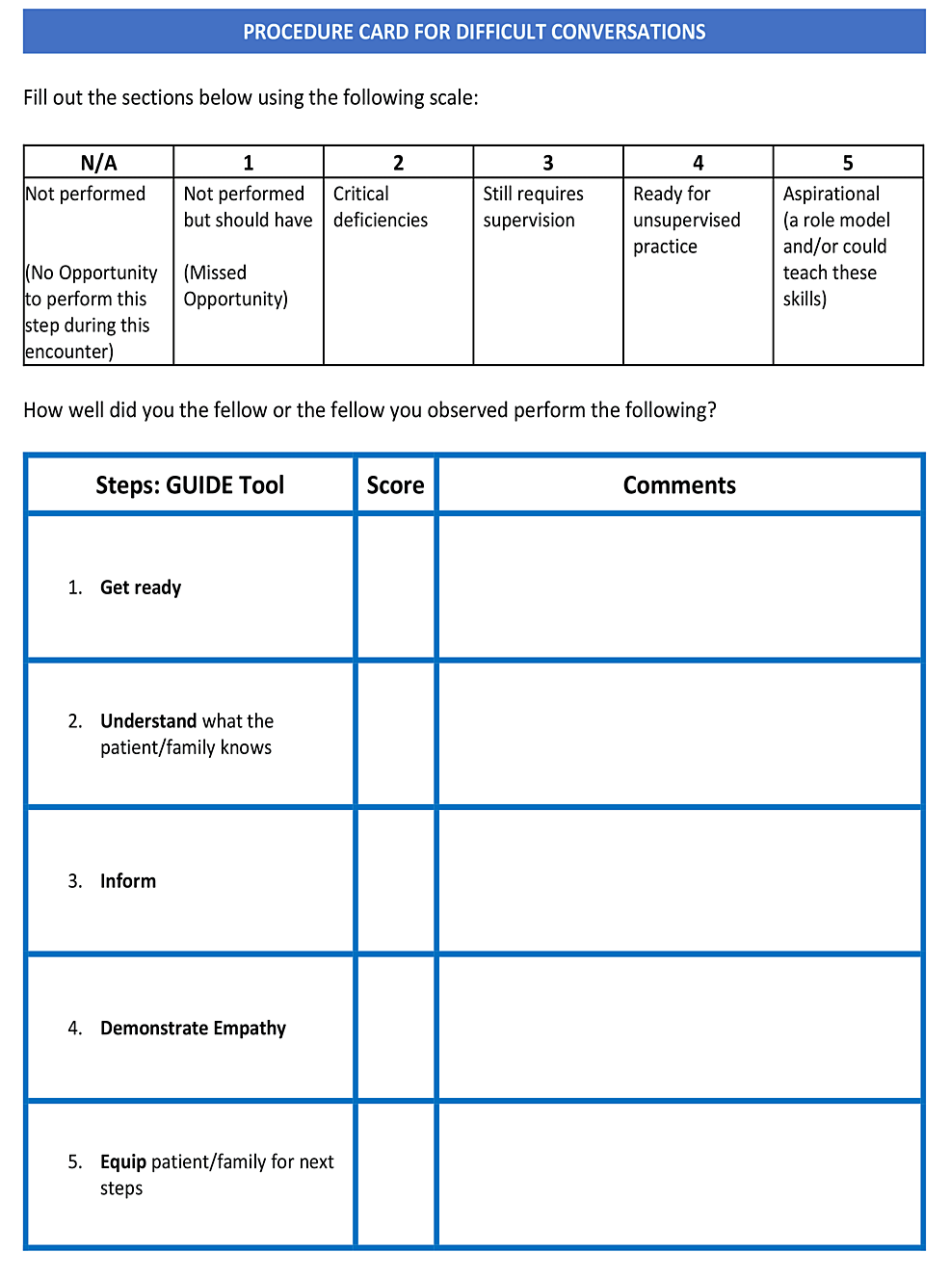
Procedure card for delivering serious news (side B) Adapted from VitalTalk [20]

One month after the simulations, all fellows participated in an interactive educational session facilitated by two of the study investigators who are also trained VitalTalk faculty [20]. During this session, fellows reviewed their skills to deliver difficult news and respond to emotion and practiced these skills through role-play. Following the session, participants were introduced to a procedure card (Figures 1, 2), adapted from VitalTalk [20] with permission and designed by the investigators.

The card, including the GUIDE (Get Ready, Understand, Inform, Demonstrate Empathy, and Equip) communication framework, served as a prompt for the necessary skills and steps in this procedure. In addition, the procedure card served as a tool to promote bedside coaching and feedback in real-time [20]. Concurrently, investigators introduced this study and procedure card to 25 attending physician faculty from the participants’ respective divisions. This session highlighted key communication skills and ways to coach fellows at the bedside before, during, and after a communication procedure. Coaching instructions were modified from VitalTalk [20] coaching techniques and distributed to attending physicians.

Phase 3: use of the procedure card

During phase 3 of the study, participants were asked to perform six procedures (delivering serious news) with actual patients and families during a span of six months. Participants were assigned a study identification number enabling completed procedure cards to be de-identified. Participants were asked to meet with supervising faculty immediately prior to the procedure to review key elements of the procedure card. Fellows were told to use the procedure card as a prompt or reference prior to and during the procedure to serve as a reminder of the steps and skills required to navigate these conversations. After the difficult conversation, faculty were expected to debrief the encounter with the fellow. In addition, both the participant and the faculty member rated the participant’s performance and skill with the procedure by utilizing a 1-5 Likert scale.

Phase 4: reassessing performance using simulation

Once all participants completed their six-month period of procedure card use, they completed the same self-efficacy survey from the start of the study. Participants took part in a final simulation session in which they had a difficult conversation with a parent portrayed by an actor. Following the simulation, study investigators and actors assessed the performance. The data garnered from this second simulation session was compared with each participant’s baseline performance level. A debrief followed each simulation session during which the participant’s strengths and opportunities for improvement were reviewed. At the end of the session, participants completed an end-of-study survey exploring the barriers to and successes of the study. The survey used a 1-5 Likert scale, with 1 indicating "strongly disagree" and 5 indicating "strongly agree."

## Results

Due to the limited number of fellows, this was carried out as a convenience sample and a descriptive study detailing pre- and post-intervention analyses. Data were analyzed using paired samples t-tests as appropriate. Of the 17 eligible participants, 11 were enrolled in the study: 7/7 critical care, 2/2 hematology/oncology, and 2/8 neonatology fellows. One neonatology fellow was excluded from the analysis as the fellow did not complete phase 3 or phase 4 of the study. Thus, only 10 out of 17 eligible participants completed the study. Five participants were in their second year of fellowship, and five were first-year fellows. Of note, 6/10 participants (60%) completed the procedure cards.

A statistically significant increase in self-efficacy was reported by all fellows regarding the skills of delivering difficult news, conducting a family conference, and responding to emotions (Table 1).

**Table 1:** Self-efficacy survey results Likert scores shown as mean (SD) Pre: pre-intervention. Post: post-intervention. SD: standard deviation

Communication procedure	Total (n=10)			No procedure cards (n=4)			Procedure cards (n=6)		
	Pre	Post	P-value	Pre	Post	P-value	Pre	Post	P-value
Deliver difficult news	3.0 (0.67)	4.1 (.57)	0.001	2.5 (0.58)	4.0 (0)	0.14	3.33 (0.52)	4.2 (0.75)	0.42
Conduct family conference	2.5 (0.53)	3.6 (1.11)	0.001	2.5 (0.58)	3.75 (0.5)	0.15	2.5 (0.55)	3.5 (1.38)	0.41
Respond to emotions	3.4 (0.7)	4.2 (.42)	0.003	2.75 (0.5)	4.0 (0)	0.15	3.83 (0.41)	4.33 (0.52)	0.76
Discuss prognosis	2.9 (0.57)	3.7 (0.67)	0.22	3.25 (0.5)	3.5 (0.58)	0.638	2.67 (0.52)	3.83 (0.75)	0.13
Discuss various treatment options including palliative care	2.6 (0.84)	3.4 (0.52)	0.11	2.75 (0.5)	3.0 (0)	0.391	2.5 (1.05)	3.67 (0.52)	0.13
Discuss code status	2.5 (0.85)	2.9 (0.57)	0.223	2.75 (0.96)	3.0 (0)	0.638	2.33 (0.82)	2.83 (0.75)	0.29
Facilitate conversation about end-of-life care	2.3 (0.67)	3.0 (0.94)	0.66	2.25 (0.5)	2.75 (0.5)	0.182	2.33 (0.82)	3.17 (1.17)	0.185

There were no differences in self-efficacy scores between those who completed the procedure cards and those who did not (Table 2). The data show the investigators’ simulation-based assessment of performance revealing an overall statistically significant improvement in the fellows’ skill in delivering news and responding to emotions. However, subset analysis revealed that this improvement was identified only in those who filled out the procedure cards.

**Table 2:** Investigator assessment during simulation Likert scores shown as mean (SD) Pre: pre-intervention. Post: post-intervention. SD: standard deviation

Steps	Total (n=10)			No procedure cards (n=4)			Procedure cards (n=6)		
	Pre	Post	P-value	Pre	Post	P-value	Pre	Post	P-value
Get ready	3.5 (0.71)	3.8 (0.42)	0.81	3.5 (0.58)	3.75 (0.25)	0.391	3.5 (0.84)	3.83 (0.41)	0.175
Understand what the family knows	3.11 (1.05)	2.78 (1.2)	0.524	2.5 (1.29)	1.75 (0.96)	0.547	3.6 (0.55)	3.5 (0.55)	1
Inform	2.5 (0.71)	3.9 (0.57)	0.0001	2.25 (0.5)	3.5 (0.58)	0.015	2.67 (0.82)	4.17 (0.41)	0.001
Demonstrate empathy	2.7 (0.67)	3.3 (0.95)	0.005	2.5 (0.58)	2.75 (0.96)	0.391	2.83 (075)	3.67 (0.82)	0.004
Equip patient/family for the next steps	2.67 (1.21)	3.17 (1.33)	0.296	1.5 (0.71)	2.0 (1.41)	0.5	3.25 (0.96)	3.75 (0.96)	0.495

Actors’ assessments also demonstrated statistically significant improvement in the skill of delivering news overall (Table 3).

**Table 3:** Actor assessment during simulation Likert scores shown as mean (SD) *Unable to calculate the p*-*value because the standard error of the difference is 0 Pre: pre-intervention. Post: post-intervention. SD: standard deviation

Steps	Total (n=10)			No procedure cards (n=4)			Procedure cards (n=6)		
	Pre	Post	P-value	Pre	Post	P-value	Pre	Post	P-value
Get ready	3.5 (0.71)	4.0 (0)	0.52	3.0 (0)	4.0 (0)	*	3.83 (0.76)	4.0 (0)	0.611
Understand what the family knows	3.67 (1.12)	2.78 (1.48)	0.184	3.25 (1.5)	1.5 (1.0)	0.0001	4.0 (0.71)	3.8 (0.84)	0.621
Inform	2.6 (0.7)	3.9 (0.32)	0.0001	2.25 (0.5)	3.75 (0.5)	0.667	2.83 (0.75)	4.0 (0)	0.013
Demonstrate empathy	3.1 (0.99)	3.4 (0.84)	0.081	2.75 (0.5)	3.0 (0.82)	0.184	3.33 (1.21)	3.67 (0.82)	0.175
Equip patient/family for the next steps	2.5 (1.52)	3.17 (0.98)	0.175	2.5 (0.71)	2.5 (0.71)	^a^	2.5 (1.91)	3.5 (1.0)	0.182

In a post-intervention survey, fellows reported the perceived barriers to and successes of the study (Table 4).

**Table 4:** End-of-study survey results GUIDE: Get Ready, Understand, Inform, Demonstrate Empathy, Equip. SD: standard deviation

Item	Mean Likert score (SD)
Easy to complete a procedure card	3.29 (1.254)
GUIDE framework beneficial	4.13 (0.991)
Attending facilitation beneficial	4.5 (0.535)
Enough time to fill out cards	3.56 (1.424)
Easy to remember to fill out cards	2.11 (1.167)
Clear process to fill out cards	4.10 (0.738)
Faculty available	2.89 (1.054)
Faculty willing to participate	3.89 (0.782)
Comfortable approaching faculty	4.00 (0.707)
Ample appropriate encounters	3.30 (1.059)

## Discussion

This pilot study describes a novel mixed-methods communication curriculum integrating didactic, role-play, bedside faculty coaching, and a procedure-card prompt designed to reinforce skills in delivering difficult news. After completing this curriculum, all pediatric subspecialty fellows reported increased confidence in delivering difficult news. Additionally, fellows demonstrated improvement in communication skills as per the observed assessment after six months, specifically in terms of 1) delivering serious news clearly and 2) responding to emotions.

Although there have been various studies looking at simulation and role-play to improve communication skills [9-19,21,22], this is the first to describe the use of a procedure card and bedside coaching. Interestingly, improvement in the skills required to respond to emotions was demonstrated only by those participants who were able to use the procedure card with bedside coaching, suggesting that it is a more complex skill that requires deliberate practice and coaching to master.

While this pilot curriculum overall demonstrated sustained effectiveness over a six-month period in improving fellows’ communication skills, there were several limitations to the study. Logistically, it was difficult for fellows to participate in phase 2 of the study due to conflicting schedules. Despite reports of the faculty’s willingness to participate in bedside coaching, no fellow was able to complete the expected number of procedure cards and coached-communication encounters. Possible contributing factors included lack of perceived opportunity to have difficult conversations, concerns about being directly observed by supervising faculty, difficulty remembering to fill out cards, or decrease in patient volume, and restrictions on the number of participants permitted in a family meeting imposed during the initial phase of the coronavirus pandemic.

Additional limitations such as the small sample size at a single institution preclude the generalizability of the curriculum. What remains clear is that the participants who completed the encounter with the card and coaching had a significant improvement in the scores on "responding to emotions." However, due to the limited sample size, it is difficult to ascertain what other variables contributed to the results. It is possible that the trainees' skills improved with continued practice accompanied by both formal training via this study and bedside practice that occurred within the framework of their fellowship training. This pilot could not control for any additional informal training and feedback the fellows may have received during the designated time period. There were also limitations in reliable coaching at the bedside. Despite faculty receiving training in the communication framework, evaluation scale, and coaching guide at the bedside, there was likely variability in coaching and feedback between faculty members.

We plan to modify this curriculum to enhance its feasibility for more widespread use. Given the limitations of faculty availability and variability in coaching, for future studies, we hope to re-focus on the efficacy of the procedure card alone to reinforce the steps and techniques needed to skillfully conduct a difficult conversation.

## Conclusions

Teaching the procedure of delivering serious news should utilize the methods clinicians rely on to learn other vital procedures: following a stepwise framework, engaging in deliberate practice, and incorporating feedback to achieve proficiency. In this small pilot study of pediatric subspecialty fellows, the deliberate use of coaching at the bedside with a procedure-card prompt was effective at enhancing and retaining communication skills. This pilot should help inform future research focused on communication training in medicine as well as the modifications needed to enhance the curriculum's feasibility.
